# The risk of uterine rupture in trial of labor after cesarean in grand multiparous parturients

**DOI:** 10.1002/pmf2.70322

**Published:** 2026-06-11

**Authors:** Aviya Hetzroni, Elyasaf Bitton, Shulamit Hakuone, Yael Yekel, Ron Rosenberg, Hila Hochler

**Affiliations:** ^1^ Department of Obstetrics and Gynecology Laniado Medical Center Netanya Israel; ^2^ Adelson School of Medicine Ariel University Ariel Israel

**Keywords:** grand multiparity, labor augmentation, labor induction, parity, trial of labor after cesarean, uterine rupture, vaginal birth after cesarean

## Abstract

**Background:**

Previous cesarean delivery is the main risk factor for subsequent uterine rupture. This study aimed to determine whether grand multiparity (≥5th delivery) elevates the risk for uterine rupture in trial of labor after cesarean (TOLAC).

**Study Design:**

Retrospective study including all TOLACs with vertex presentation, from 2013 to 2024 in a university‐affiliated medical center. The study groups were as follows: (1) grand multiparous parturients (current delivery ≥5), (2) parturients at their third–fourth delivery, and (3) parturients at their second delivery. The primary outcome was uterine rupture rate. Secondary outcomes were obstetric and neonatal complications.

**Results:**

A total of 8410 TOLACs were recorded during the study period, including 2516 grand multiparous parturients, 2695 women at their third–fourth delivery, and 3199 parturients at their second delivery. Grand multiparous parturients were older (*p* < 0.001), had higher birthweights (*p* < 0.001), and had less preterm deliveries (*p* = 0.003). Induction and augmentation of labor were conducted in 15% and 17% of grand multiparous parturients, respectively, 11.7% and 15.2% in third–fourth deliveries, and 13.9% and 29% in second deliveries (*p* = 0.001). Incidence of uterine rupture was lowest among grand multiparous women (0.2% for grand multiparous, 0.6% for third–fourth delivery, and 0.9% for second delivery, *p* = 0.002). A multivariable model adjusted for maternal age, birthweight, induction, and augmentation revealed that grand multiparity was not associated with an increased risk of uterine rupture in TOLAC, compared to both second (adjusted odds ratio [aOR], 0.16; *p* < 0.01) and third–fourth deliveries (aOR, 0.27; *p* = 0.014). Re‐analysis restricted to women with parity ≥6, compared to lower‐parity women, yielded identical results.

**Conclusions:**

Grand multiparity does not appear to be associated with an increased risk of uterine rupture in TOLAC.

## 1 INTRODUCTION

Trial of labor after cesarean (TOLAC) is widely promoted in order to reduce repeat cesarean deliveries and their associated maternal and obstetric risks [1, 2, 3]. Uterine rupture represents a severe complication of TOLAC and remains a major determinant of counseling and intrapartum management decisions, emphasizing the need for careful candidate selection [4, 5].

Earlier literature proposed an association between grand multiparity and uterine rupture during labor in women with both scarred and unscarred uteri. However, this view was largely informed by older observational studies with limited sample sizes [6, 7, 8]. Recent studies have called this assumption into question, indicating that higher parity does not increase the risk of uterine rupture among women undergoing TOLAC [9, 10, 11].

Despite these reassuring observations, evidence regarding uterine rupture risk in grand multiparous women undergoing TOLAC remains limited, particularly in the presence of labor induction and augmentation.

Our goal is to evaluate the association between grand multiparity and uterine rupture among women undergoing TOLAC in a large cohort, delivering at a high‐resource medical center that routinely employs labor induction in this population.

## 2 METHODS

We conducted a retrospective cohort study at a university‐affiliated medical center. Our Labor and Delivery unit is characterized by a high annual delivery volume exceeding 8000 deliveries, with grand multiparous women constituting 29.9% of the study population. Notably, our patient population is drawn from nonselected, ethnically and sociodemographically diverse populations of the medical center catchment area.

The perinatal database contains information recorded by obstetricians during and immediately after delivery. Coding for the medical diagnoses was performed according to the International Classification of Disease 9 (ICD‐9) codes. Experienced medical secretaries routinely review the information in order to ensure completeness and accuracy of the data. Additionally, in all cases of uterine rupture, we manually reviewed all available medical records, including admission records, labor and delivery cards, operation reports, and discharge letters. These procedures help to assure maximal completeness and accuracy of our databases.

Following approval from the institutional ethics committee, which exempted informed consent, electronic medical records of all deliveries between 2013 and 2024 were reviewed. The analysis included all cases of trial of labor after cesarean (TOLAC) meeting the following inclusion criteria: parturient with singleton pregnancies with one prior cesarean delivery (CD) who underwent TOLAC, with a live fetus in vertex presentation, gestational age ≥24+0 weeks, and no contraindications for vaginal delivery.

Women were excluded in cases of more than one previous CD, elective CD, emergency CD without an attempted trial of labor, gestational age below 24 weeks, non‐vertex presentation, or multiple gestation.

Study groups were defined according to parity:

1. grand multiparous women (current delivery ≥5) comprised the study group;
2. women presenting for their third or fourth delivery;
3. women presenting for their second delivery.

All records were anonymized and de‐identified in accordance with institutional ethical standards prior to analysis.

The primary outcome was uterine rupture rate (ICD‐9 code 665.1). This code included only cases of separation of all uterine wall layers. Cases of dehiscence of the uterine scar were not classified as uterine rupture.

Secondary outcomes included maternal and neonatal complications.

Two low‐dose oxytocin protocols were used for induction and augmentation of labor: a standard low‐dose protocol (starting at 1 mU/min with increments of 1 mU/min every 20 min), which was administered to women with a prior CD and parity up to five, and a very low‐dose protocol (starting at 0.5 mU/min with increments of 0.5 mU/min every 20 min), which was reserved for women with a prior CD and parity of six or greater.

Counseling regarding TOLAC was performed according to institutional practice and was not modified based on parity. All patients were counseled regarding the potential risks and benefits of TOLAC, including the increased risk of uterine rupture associated with the use of oxytocin for labor induction or augmentation.

### 2.1 Statistical analysis

Categorical variables were summarized as frequencies and percentages. The distribution of continuous variables was assessed using histograms. Normally distributed continuous variables were reported as mean and standard deviation (SD), whereas non‐normally distributed variables were presented as median and interquartile range (IQR). The Chi‐square test was applied to compare categorical variables among the three parity groups. Continuous variables were compared using either the Welch ANOVA or the Kruskal–Wallis test, as appropriate.

A multivariable logistic regression model was used to examine the association between uterine rupture and parity group, adjusting for maternal age, birthweight, induction of labor, and augmentation of labor. All statistical tests were two‐tailed, and a *p*‐value < 0.05 was considered statistically significant. Statistical analyses were performed using SPSS software, version 29.0.2 (IBM Corp.).

## 3 RESULTS

During the study period, there were 90,819 deliveries at our medical center. A total of 8410 TOLACs met the inclusion criteria and were included in the analysis. Of those, 3199 (38%) were women at their second delivery, 2695 (32%) were women at their third–fourth delivery, and 2516 (29.9%) were grand multiparous women. Clinical and demographic characteristics of the groups are presented in Table 1. Additional characteristics of women with parity ≥6 are presented in Table S1. As anticipated, maternal age differed significantly across parity groups, increasing progressively from 30.4 ± 5.1 years among women at their second delivery to 31.9 ± 4.7 years in those at their third–fourth delivery, and 35.6 ± 4.2 years among grand multiparous women (*p* < 0.001) (Table 1). Grand multiparous women had higher birthweights (*p* < 0.001) and fewer preterm deliveries (*p* = 0.003) (Table 1). Induction and augmentation of labor were conducted in 15% and 17% of grand multiparous women, respectively, in 11.7% and 15.2% of women at their third–fourth deliveries, and in 13.9% and 29% of women at their second deliveries (*p* = 0.001) (Table 1).

**TABLE 1:** Demographic characteristics.

	Para 2	Para 3–4	Para 5+	
	*n* = 3199	*n* = 2695	*n* = 2516	
	%(38)	%(32)	%(29.9)	*p* value
Maternal age, years SD	30.42 ± 5.13	31.88 ± 4.7	35.56 ± 4.18	<0.001
Gestational age, weeks SD	39.94 ± 1.58	39.92 ± 1.38	40.08 ± 1.46	<0.001
Birthweight, g SD	3.3 ± 0.47	3.36 ± 0.47	3.44 ± 0.49	<0.001
Parity	2.00 ± 0.00	3.36 ± 0.48	7.08 ± 2.03	
Preterm delivery (<37weeks)	3.8 (120)_a_	3.0 (81)_a,b_	2.2 (55)_a_	0.003
GDM	6.7 (214)_a_	7.7 (208)_a_	9.7 (244)_b_	<0.001
Pregestational diabetes	1.0 (31)_a_	0.3 (9)_b_	0.8 (21)_a_	<0.001
Hypertension	1.6 (51)_a_	0.9 (23)_b_	1.3 (33)_a,b_	0.04
Induction of labor	13.9 (445)_a_	11.7 (314)_b_	15 (378)_a_	0.001
Augmentation of labor	29 (928)_a_	15.2 (410)_b_	17.0 (428)_b_	<0.001

*Note*: Categories sharing the same subscript letter do not differ significantly from each other (*p* > 0.05). Categories with different subscript letters differ significantly (*p* < 0.05).

Uterine rupture occurred in 49 (0.6%) cases overall, with a statistically significant decreasing trend across parity groups: 0.9% in women at their second delivery, 0.6% in those at their third–fourth delivery, and 0.2% among grand multiparous women (*p* = 0.002) (Table 2).

**TABLE 2:** Perinatal outcomes.

	Para 2	Para 3–4	Para 5+	
	*n* = 3199	*n* = 2695	*n* = 2516	
	%(38)	%(32)	%(29.9)	*p* value
Uterine rupture	0.9 (29)_a_	0.6 (15)_a_	0.2 (5)_b_	0.002
Cesarean delivery	31.2 (996)_a_	11 (297)_b_	7.8 (196)_c_	<0.001
Operative delivery	20.7 (661)_a_	7.2 (194)_b_	4.1 (103)_c_	<0.001
Peripartum hysterectomy	0.0 (0)	0.0 (1)	0.0 (1)	0.529
PPH	5.2 (165)	4.2 (114)	4.5 (112)	0.206
Blood transfusion	3.9 (124)_a_	1.6 (44)_b_	1.8 (46)_b_	<0.001
Placental abruption	1.3 (43)_a_	1.2 (31)_a_	0.6 (16)_b_	0.032
Shoulder dystocia	0.3 (9)_a_	0.7 (19)_b_	1.4 (34)_c_	<0.001
Apgar 5 min <7	0.7 (23)	0.4 (11)	0.5 (12)	0.234

*Note*: Categories sharing the same subscript letter do not differ significantly from each other (*p* > 0.05). Categories with different subscript letters differ significantly (*p* < 0.05).

Abbreviation: PPH, postpartum hemorrhage.

Among the 49 cases of uterine rupture, prior vaginal birth after cesarean (VBAC) status was examined descriptively. Twenty‐nine cases occurred in women at their second delivery, for whom prior VBAC was not applicable. Among women at their third delivery (*n* = 7), three had a history of prior VBAC. Among those at their fourth delivery (*n* = 8), two had a prior VBAC history, including one with a single prior VBAC and one with two prior VBACs. Among women at their fifth delivery (*n* = 5), only one had a history of prior VBAC (with two prior VBACs), while the remaining cases had no prior VBAC.

CD was performed in 1489 (17.7%) cases, with a significant decrease across parity groups: 996 (31.1%) among women at their second delivery, 297 (11.0%) among those at their third–fourth delivery, and 196 (7.8%) among grand multiparous women (*p* < 0.001) (Table 2).

Operative vaginal delivery was also significantly more common among women at their second delivery (20.7%), compared with those at their third–fourth (7.2%) and grand multiparous deliveries (4.1%) (*p* < 0.001) (Table 2).

Peripartum hysterectomy was exceptionally rare and occurred in only 2 (0.02%) cases across the entire cohort, with no significant difference between parity groups (*p* = 0.53) (Table 2). One of the cases occurred in the third–fourth delivery group, and the other among grand multiparous.

Postpartum hemorrhage (PPH) occurred in 391 (4.6%) women overall, with comparable rates across the groups (Table 2). The need for blood transfusion decreased significantly with increasing parity, observed in 124 (3.9%), 44 (1.6%), and 46 (1.8%) cases, respectively (*p* < 0.001) (Table 2).

Low Apgar scores (<7 at 5 min) were uncommon, recorded in 46 (0.5%) neonates, with no significant difference between groups (*p* = 0.23) (Table 2).

Multivariable model adjusted for maternal age, birthweight, induction and augmentation revealed that grandmultiparity was not associated with an increased risk of uterine rupture in TOLAC, compared to both second (aOR, 0.16; *p* < 0.01) and third–fourth deliveries (aOR, 0.27; *p* = 0.014) (Table 3). Additional regression analyses are presented in Table S2.

**TABLE 3:** Regression model for the association between uterine rupture and grandmultiparity, maternal age, fetal weight, induction, and augmentation.

	Adjusted OR	95% CI	*p* value
Para 5+	Reference	—	0.001
Para 2	6.235	2.28–17.02	<0.001
Para 3–4	3.669	1.30–10.38	0.014
Maternal age	1.045	0.99–1.10	0.127
Fetal weight	1.953	1.06–3.60	0.032
Induction of labor	2.339	1.16–4.71	0.017
Augmentation	1.246	0.63–2.44	0.522

Abbreviations: CI, confidence interval; OR, odds ratio.

In the total cohort, both induction of labor (aOR, 2.34; 95% confidence interval [CI], 1.16–4.71; *p* = 0.017) and higher fetal birthweight (aOR, 1.95; 95% CI, 1.06–3.60; *p* = 0.032) were independently associated with uterine rupture (Table 3). Despite the large sample size, the rarity of uterine rupture within each parity group precluded stratified analysis of the association of uterine rupture and induction of labor according to parity.

In an additional subgroup analysis restricted to women who did not undergo induction or augmentation of labor, grand multiparous women demonstrated lower rates of uterine rupture compared with both second‐ and third–fourth‐delivery groups. However, no significant difference was observed between women at their second delivery and those at their third–fourth deliveries.

In this subgroup, no uterine rupture events were observed among women with parity ≥5, precluding the performance of a multivariable analysis within this group. Additional subgroup analyses are presented in Table S3.

Maternal age and labor augmentation were not significantly associated with uterine rupture risk (*p* = 0.13 and *p* = 0.52, respectively) (Table 3).

Notably, re‐analysis restricted to women with parity ≥6, compared to lower‐parity women, according to the same two control groups, yielded identical results (Table S4).

## 4 DISCUSSION

In our large retrospective cohort study including 8410 women undergoing TOLAC, we found that grand multiparity was not associated with an increased rate of uterine rupture compared with lower parity groups.

The association between grand multiparity and uterine rupture risk during TOLAC has been controversial. Grand multiparity was proposed as a potential risk factor for uterine rupture in women with or without a previous CD, largely based on data from older and relatively small studies from low‐resource settings [6, 7, 8]. However, more recent studies have challenged this view, indicating that increasing parity does not increase the risk of uterine rupture among women with a previous CD [9, 12, 13]. These conclusions are derived from methodologically robust studies characterized by large sample sizes, advanced healthcare settings, multivariable adjustment for key confounders, and multicenter designs. Additionally, several investigations were population‐based and included nonselected cohorts, enhancing external validity and reflecting routine clinical practice [9, 11, 14, 15].

Overall, current evidence indicates that, with respect to parity, only the second delivery is associated with an increased risk of uterine rupture during TOLAC, whereas grand multiparity is not. This pattern may be driven by the strong protective effect of a prior vaginal delivery, which has been consistently identified as the most important independent factor associated with reduced uterine rupture risk during TOLAC [3, 14, 16, 17]. An early study by Zelop et al. demonstrated that the risk of uterine rupture during TOLAC was fivefold higher among women attempting vaginal birth at their second delivery compared to women with prior vaginal births [18]. However, parity was not stratified in that cohort, and grand multiparous women were not specifically analyzed, limiting the applicability of these findings to high‐parity populations such as ours. Large multicenter data from Landon et al. further identified prior vaginal delivery, particularly prior VBAC, as an independent protective factor for uterine rupture in multivariable analysis [17]. Lopian et al. demonstrated that grand multiparity was not associated with an increased risk of uterine rupture during TOLAC, whereas women with no prior vaginal delivery had the highest rates of uterine rupture and adverse outcomes [9].

Labor induction and augmentation are a major concern, as they increase the risk of uterine rupture [19, 20, 21, 22]. Therefore, not all medical centers employ them in women with a previous CD.

In our cohort, induction and augmentation of labor were commonly employed during TOLAC, including among grand multiparous women (Table 1).

Counseling regarding TOLAC was not modified according to parity at our institution, reducing the likelihood that differences in patient selection contributed to the observed findings.

Importantly, induction and augmentation protocols in grand multiparous patients were low dose. In our cohort, only induction of labor was independently associated with uterine rupture. Unfortunately, despite our large cohort, the small number of uterine rupture cases in each parity group precluded statistical analysis of the association between induction of labor and uterine rupture according to parity.

In a subgroup analysis restricted to women who did not undergo induction or augmentation of labor (Table 4), grand multiparous women continued to demonstrate lower rates of uterine rupture compared with both second‐ and third–fourth‐delivery groups, whereas no significant difference was observed between women at their second delivery and those at their third–fourth deliveries. In this subgroup without induction or augmentation, no uterine rupture events were observed among women with parity ≥5, which precluded the performance of a multivariable analysis.

**TABLE 4:** Subgroup analysis of uterine rupture rates according to parity among women undergoing TOLAC without labor induction or augmentation.

	Para 2	Para 3–4	Para 5+	*p* value
	*n* = 2134	*n* = 2139	*n* = 1930	
	(34.4%)	(34.5%)	(31.3%)	
Uterine rupture	0.7 (15)_a_	0.6 (13)_a_	0.2 (0)_b_	0.002

*Note*: Categories sharing the same subscript letter do not differ significantly from each other (*p* > 0.05). Categories with different subscript letters differ significantly (*p* < 0.05).

In the literature, data addressing induction and augmentation during TOLAC in grand multiparous women are sparse. Available cohort studies, including multicenter data, suggest that grand multiparity does not confer an additional risk of uterine rupture beyond the baseline risk associated with TOLAC, including cases involving labor induction [11, 23, 24]. A multicenter study by Hochler et al. specifically addressed the safety of labor induction and augmentation during TOLAC in grand multiparous women. This cohort included 1304 grand multiparous parturients who attempted TOLAC with labor induction or augmentation. The data demonstrated low uterine rupture rates that were comparable across parity groups [11]. Despite frequent labor induction and augmentation administration among the grand multiparous women in our cohort, the low incidence of uterine rupture suggests that, with proper monitoring and low‐dose protocols, both spontaneous and induced TOLAC can be safely offered to eligible grand multiparous patients [4].

An important methodological consideration underlying these findings relates to the induction and augmentation protocols applied across studies. Several studies reporting no increased risk of uterine rupture with induction or augmentation during TOLAC in grand multiparous women explicitly describe the use of any oxytocin regimens in scarred uteri, and particularly grand multiparity. A previous multicenter cohort [11] applied induction and augmentation using a low‐dose oxytocin protocol starting at 1 mU/min with gradual increments, and uterine rupture rates remained low and comparable across parity groups [11]. Other cohort studies supporting the overall safety of TOLAC in grand multiparous women did not consistently report protocol details regarding oxytocin dosing or induction methods, limiting direct comparisons between induction strategies [9, 10].

Beyond the risk of uterine rupture, counseling women considering TOLAC should also consider the overall likelihood of successful vaginal birth, as prior studies have consistently reported favorable TOLAC outcomes among grand multiparous women [9, 12, 13, 25]. Reported TOLAC success rates in this population range from 88% to over 90%, based on cohorts specifically evaluating grand multiparous women [9, 13, 23, 24]. In line with these reports, our findings further support the notion that grand multiparous women represent a subgroup with a high probability of successful TOLAC, reinforcing the clinical rationale for offering a trial of labor in this population.

Taken together, the combination of low uterine rupture rates and high likelihood of vaginal delivery success provides a strong rationale for offering TOLAC to grand multiparous women.

Our study is strengthened by the large cohort of 8410 women undergoing TOLAC and by the substantial proportion of grand multiparous women, a group that remains underrepresented in much of the literature. Conducting the study within a single center allowed uniform intrapartum management. In addition, the study was conducted in a country with an advanced healthcare system, and all cases of uterine rupture were manually reviewed in the medical records to ensure diagnostic accuracy.

Several limitations should be considered when interpreting our findings. The retrospective nature of the study with its inherent limitations. In particular, data on prior VBAC, which is a protective factor against uterine rupture, were not available in our dataset and therefore could not be included in the multivariable analysis. In addition, information on interdelivery interval from the prior CD was unavailable. Furthermore, the indication for the prior CD was not recorded, limiting the ability to fully adjust for baseline obstetric risk. Although induction and augmentation of labor were included in the analysis, more detailed intrapartum variables such as cervical status at induction, duration of labor, and maximal oxytocin dose were not available. Moreover, women with higher parity were managed using lower‐dose oxytocin protocols, which may have contributed to the lower observed rates of uterine rupture in this group. Therefore, differences in labor management should be considered when interpreting the findings. The findings may not be fully generalizable to other settings with different induction protocols. Nonetheless, the large cohort size, standardized care environment, and consistency of results after adjustment support the robustness of our findings.

## 5 CONCLUSIONS

TOLAC in grand multiparous women is not associated with an increased risk of uterine rupture compared with women of lower parity. Together with the high VBAC success rates reported in this population, these findings suggest that TOLAC may be safely considered in appropriately selected grand multiparous women, including cases of labor induction or augmentation.

## AUTHOR CONTRIBUTIONS

Conceptualization, data collection, and analysis were performed by the authors. All authors contributed to manuscript drafting and revision and approved the final version.

## CONFLICT OF INTEREST STATEMENT

The authors declare no conflicts of interest.

## FUNDING INFORMATION

The authors received no specific funding for this work.

## Supporting information



Supporting Information

## Data Availability

The data that support the findings of this study are available from the corresponding author upon reasonable request, subject to institutional approval.
