# Supplementary material for: The risk of uterine rupture in trial of labor after cesarean in grand multiparous parturients
Source: Pregnancy (Hoboken). 2026 Jun 11;2(4):e70322. doi: 10.1002/pmf2.70322 (PMC13344267; doi:10.1002/pmf2.70322)
Supplement: Supplementary file 1 [file PMF2-2-e70322-s001.docx]

| TABLE 1 Demographic characteristics | | | | |
| --- | --- | --- | --- | --- |
| S/P CS | para 2 | para 3-5 | para 6+ | Pvalue |
|  | n=3199 | n=3379 | n=1832 |  |
|  | %(n) | %(n) | %(n) |  |
| Maternal age, years SD | 30.42±5.13 | 32.11±4.6 | 36.5±3.86 | <0.001 |
| Gestational age, wk SD | 39.94±1.58 | 39.95±1.39 | 40.08±1.49 | <0.001 |
| Birthweight, g SD | 3.3±0.47 | 3.37±0.47 | 3.45±0.5 | <0.001 |
| Parity | 2.00±0.00 | 3.37± 0.78 | 7.85±1.86 |  |
| Preterm delivery (less than 37 wk) |  |  |  |  |
| GDM | 6.7 (214)(a) | 7.5 (253)(a) | 10.9 (199)(b) | <0.001 |
| pregestational diabetes | 1.0 (31)(a) | 0.4 (12)(b) | 1.0 (18)(a) | <0.001 |
| Hypertention | 1.6 (51)(a) | 0.9 (29)(b) | 1.5 (27)(a) | 0.02 |
| Induction of labor | 13.9 (445)(a,b) | 12.4 (419)(b) | 14.9 (273)(a) | 0.03 |
| Augmentation of labor | 29 (928)(a) | 15.4 (519)(b) | 17.4 (319)(b) | <0.001 |

Each subscript letter denotes a subset of parity_2_5 categories whose column proportions do not differ significantly from each other at the .05 level.

| TABLE 2 Maternal outcomes | | | | |
| --- | --- | --- | --- | --- |
| S/P CS | para 2 | para 3-5 | para 6+ | Pvalue |
|  | n=3199 | n=3379 | n=1832 |  |
|  | %(n) | %(n) | %(n) |  |
| Uterine rupture | 0.9 (29)(a) | 0.6 (19)(a) | 0.1 (1)(b) | 0.001 |
| CS | 31.2 (996)(a) | 9.9 (333)(b) | 8.7 (160)(b) | <0.001 |
| Peripartum hysterectomy | 0.0 (0)(a) | 0.0 (1)(a) | 0.1 (1)(a) | 0.694 |
| PPH | 5.2 (165)(a) | 4.1 (139)(b) | 87 (112)(a,b) | 0.129 |
| Blood transfusion | 3.9 (124)(a) | 1.7 (57)(b) | 1.8 (33)(b) | <0.001 |
| Placental abruption | 1.3 (43)(a) | 1.0 (33)(a) | 0.8 (14)(a) | 0.124 |
| shoulder dystocia | 0.3 (9)(a) | 0.8 (27)(b) | 1.4 (26)(c) | <0.001 |
| Apgar 5 minute <7 | 0.7 (23)(a) | 0.4 (14)(a) | 0.5 (9)(a) | 0.232 |

Each subscript letter denotes a subset of parity_2_5 categories whose column proportions do not differ significantly from each other at the .05 level.

| TABLE 3 - Regression model for the association between uterine rupture and grand-multiparity, maternal age, fetal weight, induction and augmentation | | | |
| --- | --- | --- | --- |
| Parity | Adjusted OR | 95% CI | Pvalue |
| para 6+ | Reference | .-- | 0.003 |
| para 2 | 25.310 | 3.33-192.30 | 0.002 |
| para 3-5 | 14.520 | 1.91-110.43 | 0.010 |
| age | 1.054 | 0.99-1.11 | 0.069 |
| mishkal_yilod | 2.024 | 1.09-3.74 | 0.025 |
| induction_labor_YN(1) | 2.335 | 1.16-4.69 | 0.017 |
| augmentation(1) | 1.258 | 0.64-2.46 | 0.502 |

| TABLE 4 - Subgroup analysis of uterine rupture rates according to parity among women undergoing TOLAC without labor induction or augmentation | | | | |  |
| --- | --- | --- | --- | --- | --- |
|  | para 2 | para 3-5 | para 6+ | p value |  |
|  | n=2134 | n=2662 | n=1407 |  |  |
|  | (34.4%) | (42.9%) | (22.7%) |  |  |
| Uterine rupture | 0.7 (15) a | 0.5 (13) a | 0.2 (0) b | 0.009 |  |
